# When COPD meets *Aspergillus*: A treatment Odyssey and critical insights from a 76-year-old female patient’s journey: A case report

**DOI:** 10.1097/MD.0000000000042204

**Published:** 2025-04-18

**Authors:** Miaomiao Chen, Guiying Chen, Song Wu, Yaqian Zhu

**Affiliations:** a Department of Respiratory and Critical Care Medicine, Tianjin Chest Hospital, Tianjin, China.

**Keywords:** *Aspergillus flavus*, case report, COPD, FEV1, FVC, mMRC dyspnea scale

## Abstract

**Rationale:**

Chronic obstructive pulmonary disease (COPD) is a common respiratory disorder characterized by persistent limitation of airflow. Fungal infections are not uncommon in patients with COPD. However, reports on the short-term improvement in lung function in COPD patients with IgE-negative *Aspergillus* tracheobronchitis (ATB) following antifungal treatment are currently rare. Here, we described a patient whose lung function significantly improved after antifungal treatment for COPD combined with ATB, along with the diagnostic process.

**Patient concerns:**

A 76-year-old female patient with COPD presented with worsening cough, expectoration, and wheezing. Despite some relief in respiratory symptoms after empirical antibiotic and anti-inflammatory treatment, her exercise tolerance did not return to pre-illness levels.

**Diagnoses:**

Subsequently, a bronchoalveolar lavage fluid sample was sent for microbiological examination, which identified *Aspergillus flavus* (*A flavus*).

**Interventions:**

The patient was treated with voriconazole for one week as antifungal therapy.

**Outcomes:**

The patient’s subjective symptoms were further improved, and the modified British Medical Research Council dyspnea scale returned to the stable state before the exacerbation. A follow-up pulmonary function test showed an improvement of 880 mL in forced vital capacity and 620 mL in forced expiratory volume in one second.

**Lessons:**

Treatment of acute exacerbations of COPD must emphasize careful assessment of the patient’s symptoms and remain vigilant for potential coexisting ATB.

## 1. Introduction

### 1.1. Background

Chronic obstructive pulmonary disease (COPD) is a chronic, progressively advancing respiratory disease characterized by recurrent respiratory infections and acute exacerbations.^[1]^ As the condition progresses, exacerbations become more frequent, pulmonary function deteriorates progressively, and the prognosis worsens.^[2]^ Due to changes in lung structure and immunological alterations within the respiratory system, these patients are prone to fungal infections.^[3]^ Rapid and accurate identification of fungal coinfections and appropriate antifungal treatment are crucial for slowing the decline in lung function and reducing the frequency of acute exacerbations. This case report described a COPD patient who did not initially show signs of *Aspergillus* tracheobronchitis (ATB) in routine laboratory tests. Through subtle clinical symptoms and bronchoscopic examination, the diagnosis was confirmed with bronchoalveolar lavage fluid samples, and significant improvement in lung function was achieved following antifungal treatment.

### 1.2. Rationale and knowledge gap

Fungal infections are not uncommon in patients with COPD. However, reports on the short-term improvement in lung function among COPD patients with IgE-negative ATB following antifungal treatment are currently rare.

## 2. Case presentation

A 76-year-old woman with a 5-year history of COPD was admitted to Tianjin Chest Hospital on October 2, 2023. Initially treated for COPD with salmeterol and fluticasone, she had not been adhering to her medication regimen. Her symptoms, triggered by physical activity, included increased cough frequency and more white sputum. A recent chest computed tomography (CT) scan revealed patchy opacities and thickened bronchial walls. Having smoked for over 60 years, she denied other significant health issues. Her vitals included a temperature of 36.5 °C, pulse of 56 bpm, and blood pressure of 136/59 mm Hg.

**Figure 1.**
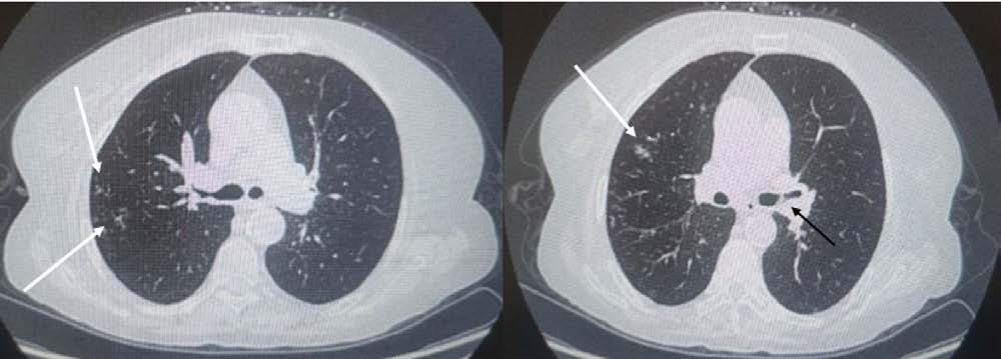
Minor patchy opacities and ground-glass opacities (white arrows) are visible in both lungs, with thickening of the bronchial walls and partial mucus impaction within the lumens (black arrow).

Following admission, routine blood tests revealed a white blood cell count of 9.04 × 10^9^/L with neutrophils comprising 79% of these cells. Tests for liver and kidney function, coagulation indicators, and serum immunoglobulins were normal. Additionally, screenings for fungal infections, including the 1,3-β-d-glucan (BG) test, *Aspergillus* galactomannan (GM) test, and *Aspergillus* IgG antibody, were all negative. An arterial blood gas analysis performed without supplemental oxygen showed a pH of 7.428, a PaCO_2_ of 38.9 mm Hg, and a PaO_2_ of 61.6 mm Hg. Sputum analysis did not reveal any fungal hyphae, spores, or acid-fast bacilli, and no pathogenic bacteria were cultured. Chest CT scan displayed minor patches and ground-glass opacities in both lungs, significant thickening of the bronchial walls, and mucus impaction in some lumens (Fig. 1). The patient was suspected to have COPD accompanied by an acute lower respiratory tract infection, which led to initiating treatment with Cefoperazone–Sulbactam (3 g every 12 hours IV), Ambroxol Hydrochloride (30 mg every 12 hours IV), and Methylprednisolone (40 mg daily IV). The steroid dosage was reduced after 3 days to 20 mg daily and was completely stopped 1 week after the start of treatment due to improvement in symptoms.

**Figure 2.**
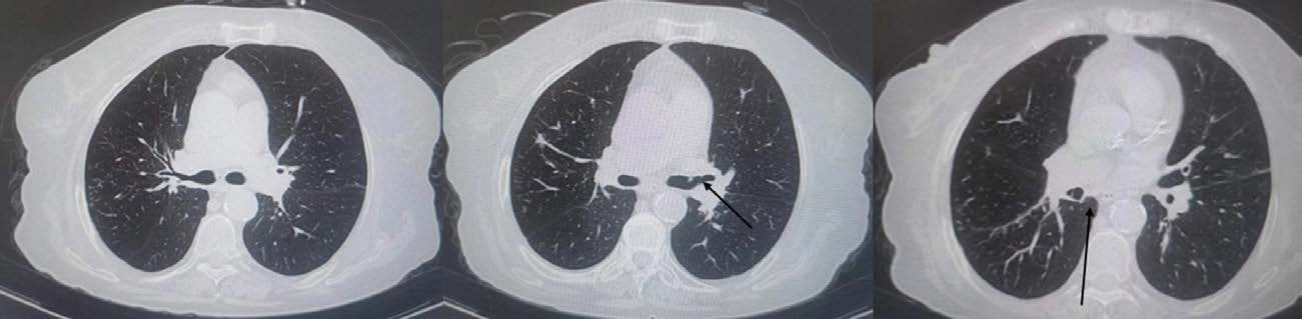
Slight improvements in the minor patchy and ground-glass opacities in both lungs compared to previous images, sputum plugs (black arrow) are still visible in some bronchial tubes.

Subsequent evaluations showed reduced white blood cell counts and slight improvements in arterial blood gas measurements; however, some persistent issues were visible on a follow-up chest CT. Notably, while some patches had slightly improved, bronchial wall thickening and mucus impaction remained largely unchanged (Fig. 2). Despite these laboratory improvements, the patient reported a decreased ability to perform activities compared to her baseline, prompting further pulmonary function tests. These revealed severely compromised lung function with a forced vital capacity (FVC) of 1.54 L and a forced expiratory volume in one second (FEV1) of 0.87 L, representing significant decreases from predicted values.

**Figure 3.**
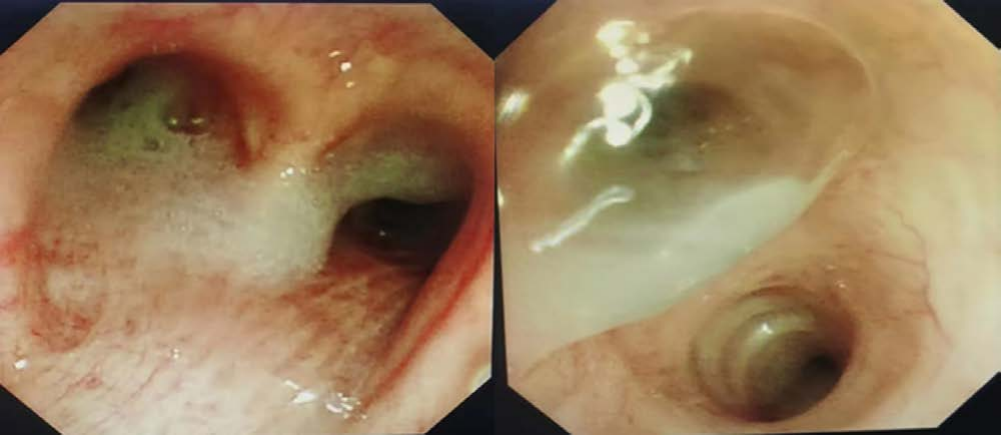
Large amounts of viscous purulent secretions in the bronchi at all levels on both sides.

Further assessments using the modified British Medical Research Council (mMRC) dyspnea scale indicated an increase in the severity of breathlessness from an mMRC score of 1 to 3. Bronchoscopy performed on October 13 found extensive purulent secretions throughout the bronchial tree (Fig. 3). Cultures from bronchoalveolar lavage fluid had tested positive for *A flavus*, confirming an *Aspergillus* infection.

**Figure 4.**
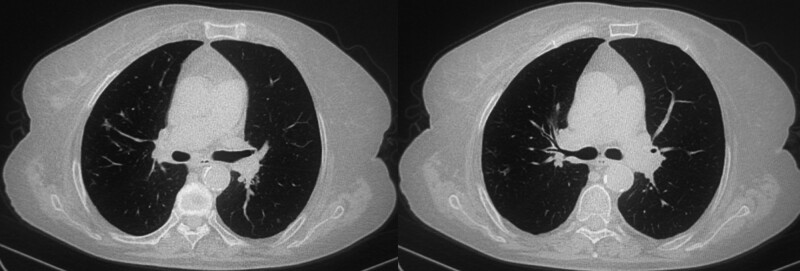
The intraluminal mucus impaction has improved significantly compared with before.

Treatment was adjusted by discontinuing previous medications and starting Voriconazole (0.3 g twice daily on day 1, followed by 0.2 g twice daily). This regimen led to noticeable improvements; the mMRC score improved to 1, and lung function measurements showed significant recovery (FVC improved by 0.88 L, FEV1 by 0.62 L) (Table 1). A chest CT 4 weeks post-discharge reflected these clinical improvements with reduced mucus impaction (Fig. 4).

**Table 1:** Changes in lung function before and after antifungal therapy.

Index	Before antifungal therapy		After antifungal treatment	
	Before inhaling bronchodilators	After inhaling bronchodilators	Before inhaling bronchodilators	After inhaling bronchodilators
FVC (L)	1.54	1.7	2.42	2.34
FEV1 (L)	0.78	0.87	1.47	1.49
FEV1/FV (%)	50.36	51.07	60.78	63.76
MEF75 (L/s)	0.89	0.97	2.22	2.03
MEF50 (L/s)	0.4	0.49	0.94	0.85

FVC = forced vital capacity, FEV1 = forced expiratory volume in one second, FEV1/FVC = the ratio of FEV1 and FVC, MEF75 = the maximal expiratory flows at 75% of vital capacity, MEF50 = the maximal expiratory flows at 50% of vital capacity.

## 3. Discussion

COPD is a common condition characterized by persistent respiratory symptoms and airflow limitation. Acute exacerbations of COPD (AECOPD) represent a significant economic burden^[4]^ and are the main cause of deteriorating quality of life.

Except bacteria and viruses, a recent study has increasingly linked fungal colonization, sensitization, and infection to AECOPD.^[3]^ However, because its clinical manifestations are easily covered by AECOPD and pneumonia-related symptoms, it is easy to be missed and misdiagnosed.

The most common fungi associated with lung infections belong to the genus *Aspergillus*, which includes invasive pulmonary aspergillosis (IPA), chronic pulmonary aspergillosis, allergic bronchopulmonary aspergillosis, *Aspergillus* sensitization, and *Aspergillus* colonization. A recent Meta-analysis showed the percentage of patients with COPD admitted to hospital with IPA varies from 1.3% to 3.9%; a mean of 2.5% was applied.^[5]^ Current study proved that intensive care unit admission, chronic heart failure, prior antibiotic and steroid treatment are the risk factors for fungal infections in COPD patients.^[6]^ It was reported that the prevalence of *Aspergillus* colonization in COPD patients is from 8.33%^[7]^ to 16.6%.^[8]^ Twenty-six percent of *Aspergillus*-colonized COPD patients developed aspergillosis.^[9]^ However, it remains uncertain whether the patient’s condition was due to the progression of preexisting *Aspergillus* colonization or a new acquisition following clinical deterioration.

ATB is a unique form of invasive *Aspergillus* (IA) disease, confined to the tracheobronchial tree with infection limited to the superficial mucosal layer of the airways.^[10]^ Common symptoms include cough, sputum production, and dyspnea. Its atypical manifestations and low detection rates often lead to delayed diagnosis and misdiagnosis. GM and BG are known markers of IA. Recent literature meta-analyses have shown a sensitivity of 0.53 and a specificity of 0.94 for serum GM for the diagnosis of IA, and the sensitivity and specificity of BG were 0.72 and 0.82, respectively.^[11]^ However, ATB rarely invades blood vessels, and serological tests for fungi are usually negative. This patient did not show significant bronchial dilation or lung infiltration, and both peripheral blood GM tests and *Aspergillus* IgG antibodies were negative. Repeated sputum cultures showed no clear evidence of fungal presence, which is a major reason why the fungal infection was not detected earlier.

Most IPA (approximately 80%) are caused by *Aspergillus fumigatus* (*A fumigatus*).^[12]^ *A flavus* is responsible for about 10% of bronchopulmonary infections.^[13]^ The pulmonary manifestations of *A flavus* are indistinguishable from those caused by *A fumigatus*^[14]^ and may include aspergilloma, IPA, or ATB. Allergic aspergillosis caused by *A flavus* is rarely reported, which might be due to interspecies variability in antigenic stimulation leading to hypersensitivity reactions. Despite experiencing symptoms of respiratory difficulty, this patient had normal levels of eosinophils and IgE in the blood, no significant bronchial dilation, and showed no signs of allergic aspergillosis.

Progressive decline in lung function is a hallmark of COPD, and repeated acute exacerbations are known to accelerate this decline. Therefore, when this patient reported a decrease in exercise capacity, it was conventionally assumed to be related to her declining lung function, especially since she felt an improvement in symptoms like coughing, sputum production, and breathing following treatment, and her blood tests showed normal white cell counts and improved oxygen levels in the blood gas analysis. This potentially misled the pulmonologist into thinking that the decrease in exercise capacity was directly related to the deterioration of her lung function. Currently, there is no definitive research specifying how much a decrease in FEV1 or FVC would need to be to affect the mMRC score. The diagnosis and treatment process of this case reminded doctors that in addition to laboratory indicators, we should also pay attention to the changes in mMRC before and after treatment to avoid misdiagnosis and missed diagnosis.

Dransfield study^[15]^ indicates that in subjects without acute respiratory exacerbations, annual FEV1 changes range from a decline of 25 mL to an increase of 5 mL, In COPD patients classified as GOLD stages 1 and 2 without exacerbations, FEV1 typically falls by 25 mL and 19 mL/yr, respectively. Each sharp FEV1 decrease correlates with acute COPD exacerbations, with the most substantial impact in GOLD stage 1, where exacerbations can lead to an additional 23 mL (and up to 87 mL in severe cases) reduction per year. Currently, there are no reports of marked changes in respiratory distress levels after such exacerbations. This patient showed marked lung function improvement after antifungal treatment for ATB, with increases of 0.88 L in FVC and 0.62 L in FEV1, illustrating that secretion blockages can significantly worsen lung function during acute COPD exacerbations. Typically, fungal bronchitis treatments improve lung function considerably, especially in cases caused by *A fumigatus*, which also see rises in total and specific IgE levels.^[16]^ However, enhancements in lung function following treatment for *A flavus*-related tracheobronchitis are rarely documented.

Although the patient’s laboratory indicators improved significantly after treatment, she experienced an increased degree of subjective breathing difficulty. Clinically, it is crucial to consider the patient’s subjective experiences and carefully analyze their imaging features. The patient’s chest CT scans showed thickened bronchial walls and mucus impaction in both lungs, suggesting a reduced airway clearance capacity. Therefore, bronchoscopic suctioning to remove viscous secretions from the airways can be effective in reducing mucus retention, controlling infection, and rapidly improving lung function.

The Infectious Diseases Society of America recommended Voriconazole is the preferred medication for IPA.^[17]^ This patient, showing copious viscous purulent secretions within the bronchi on both sides under bronchoscopy, and whose bronchoalveolar lavage fluid culture indicated *A flavus* with positive GM test, was considered to have ATB. Prompt treatment with voriconazole led to significant improvement. A follow-up chest CT 1 month later showed a reduction in airway mucus impaction.

## 4. Conclusions

Although this case involves a common disease, the treatment process was somewhat perplexing. The patient’s symptoms and laboratory indicators improved after anti-infection treatment, but the mMRC score increased compared with before this exacerbation. A definitive diagnosis was made after bronchoscopic examination, and the patient’s mMRC score decreased and FEV1 significantly improved following antifungal treatment. This serves as a reminder to clinicians to pay attention to changes in the mMRC score during acute exacerbations of COPD. It is crucial to avoid habitual thinking in the diagnosis and treatment of common diseases and to be mindful of potential complications.

## Author contributions

**Conceptualization:** Miaomiao Chen.

**Writing – original draft:** Miaomiao Chen.

**Writing – review & editing:** Miaomiao Chen, Guiying Chen, Song Wu, Yaqian Zhu.

**Project management:** Yaqian Zhu.
